# Seroprevalence of SARS‐CoV‐2 Antibodies and Associated Factors in Bamako, Mali: A Population‐Based Cross‐Sectional Study in September 2022

**DOI:** 10.1111/irv.13343

**Published:** 2024-07-23

**Authors:** Bourama Traoré, Merepen A. Guindo, Drissa Konaté, Fousseyni Kané, Nathan C. Incandela, Abdouramane Traore, Salimata Kanté, Mariam Sidibé, Bourama Keita, Fatoumata Kasse, Karamoko Tangara, Dramane Diallo, Issoufi Y. Maiga, Salif Thiam, Abdourhamane Cisse, Khatry M. Siby, Abdoul R. Dicko, Mariam Goita, Diakaridia Kone, Mamadou Diallo, Modibo Traore, Yaya I. Coulibaly, Mahamadou Diakité, Seydou Doumbia, Housseini Dolo, Saidou Balam

**Affiliations:** ^1^ International Center for Excellence in Research (ICER), Faculty of Medicine and Odontostomatology (FMOS), Faculty of Pharmacy (FAPHA) University Clinical Research Center (UCRC) at the University of Sciences, Techniques and Technologies of Bamako (USTTB) Bamako Mali; ^2^ Center for Polymers and Organic Solids, Department of Chemistry and Biochemistry University of California Santa Barbara Santa Barbara California USA; ^3^ District Health Center of Commune 4 of Bamako Minister of Health and Social Development of Mali Bamako Mali; ^4^ Hospital District Health of Commune 1 of Bamako Minister of Health and Social Development of Mali Bamako Mali; ^5^ District Health Center of Commune 6 of Bamako Minister of Health and Social Development of Mali Bamako Mali; ^6^ District Health Center of Commune 5 of Bamako Minister of Health and Social Development of Mali Bamako Mali

**Keywords:** associated factors, Bamako, Mali, SARS‐CoV‐2 antibodies, seroprevalence

## Abstract

**Background:**

The sero‐epidemiological characteristics of SARS‐CoV‐2 infections in Mali are not yet well understood. This study assessed SARS‐CoV‐2 antibody seroprevalence and factors associated with antibody responses in the general population of Bamako, the capital city and epicenter of COVID‐19, to assess the magnitude of the pandemic and contribute to control strategy improvements in Mali.

**Methods:**

A cross‐sectional survey was conducted in September 2022 to collect sociodemographic information, clinical characteristics, comorbid factors, and blood samples. ELISA was performed to determine anti‐Spike (anti‐S) and anti‐RBD antibody levels. A total of 3601 participants were enrolled in REDCap. R‐Studio was used for the statistical analysis. The chi‐squared (χ^2^) test was used to compare the proportions across different groups. Logistic regression models were used to elucidate factors associated with antibody responses.

**Result:**

The sex ratio for female‐to‐male was 3.6:1. The most representative groups were the 20–29‐year‐olds (28.9%, *n* = 1043) and the 30–39‐year‐olds (26.9%, *n* = 967). The COVID‐19 vaccine coverage among the participants was 35.8%, with vaccines from Covishield AstraZeneca (13.4%), Johnson & Johnson (16.7%), Sinovac (3.9%), and BioNTech Pfizer (1.8%). Overall, S protein and RBD antibody seroprevalences were remarkably high in the study population (98% and 97%, respectively). Factors such as youth (1–9 years old) and male sex were associated with lower SARS‐CoV‐2 antibody responses, whereas COVID‐19 vaccinations were associated with increased antibody responses.

**Conclusion:**

This serosurvey demonstrated the high seroprevalence of SARS‐CoV‐2 antibodies and highlighted the factors influencing antibody responses, while clearly underlining an underestimation of the pandemic in Mali.

## 1 Introduction

The lack of accurate estimates of the prevalence of severe acute respiratory syndrome coronavirus 2 (SARS‐CoV‐2) in Africa remains a central challenge in managing the pandemic. The virus responsible for COVID‐19 was first detected in Wuhan, China, in late 2019 [1] and was declared a global health emergency by the World Health Organization (WHO) in January 2020 [2]. The virus is primarily responsible for human and animal respiratory infections, with high morbidity and mortality rates that have negatively impacted the economic development of many countries [3, 4, 5]. Worldwide, there have been 753 million reported COVID‐19 cases and more than 6.8 million deaths in February 2023 [6]. The African continent has reported the fewest COVID‐19 cases (only 1% of global cases) [7]. Various arguments have been made to explain this, including an unfavorable climate for viral transmission, the comparatively young population of Africa, the cross‐reactivity between SARS‐CoV‐2 and other infectious diseases, and the low capacity to screen the population for infection [8, 9].

In most African countries, including Mali, there is limited published data on the seroprevalence of SARS‐CoV‐2 antibodies [10], and most epidemiological surveillance data for SARS‐CoV‐2 focuses on patients with symptoms and comorbidities and ignores the rest of the disease spectrum, including asymptomatic forms [11, 12]. In contrast, seroprevalence studies can provide key information about the prevalence of COVID‐19, as well as the scope of its impact on the general population.

In Mali, the Department of Health and Social Development reports 32,770 cumulative cases of confirmed COVID‐19, with 743 deaths, as of January 1, 2023 [13]. Most of these cases were reported in Bamako, the capital city and the epicenter of the pandemic in Mali [13, 14, 15]. To date, most serological studies in Mali have focused on healthcare workers (HCWs), and few have reported serological data from the Bamako district. Although daily national reports (from the monitoring health infrastructure) attempt to assess infection rates, they do not estimate the full scope of exposure to SARS‐CoV‐2 [15, 16, 17]. Furthermore, they do not seek to correlate antibody responses to SARS‐CoV‐2 with the sociodemographic or clinical characteristics of the general population. Overall, our objective was to estimate the seroprevalence of anti‐SARS‐CoV‐2 antibodies in the Bamako population and assess what factors contribute to the antibody responses.

## 2 Methods

### 2.1 Study Design

A cross‐sectional study was conducted in September 2022 in the general population of Bamako, Mali, as part of the WHO Unity studies [18]. Blood samples were collected to determine the anti‐Spike (anti‐S) and anti‐receptor binding domain (anti‐RBD) antibody levels in ELISA.

### 2.2 Population and Site of Study

**FIGURE 1:**
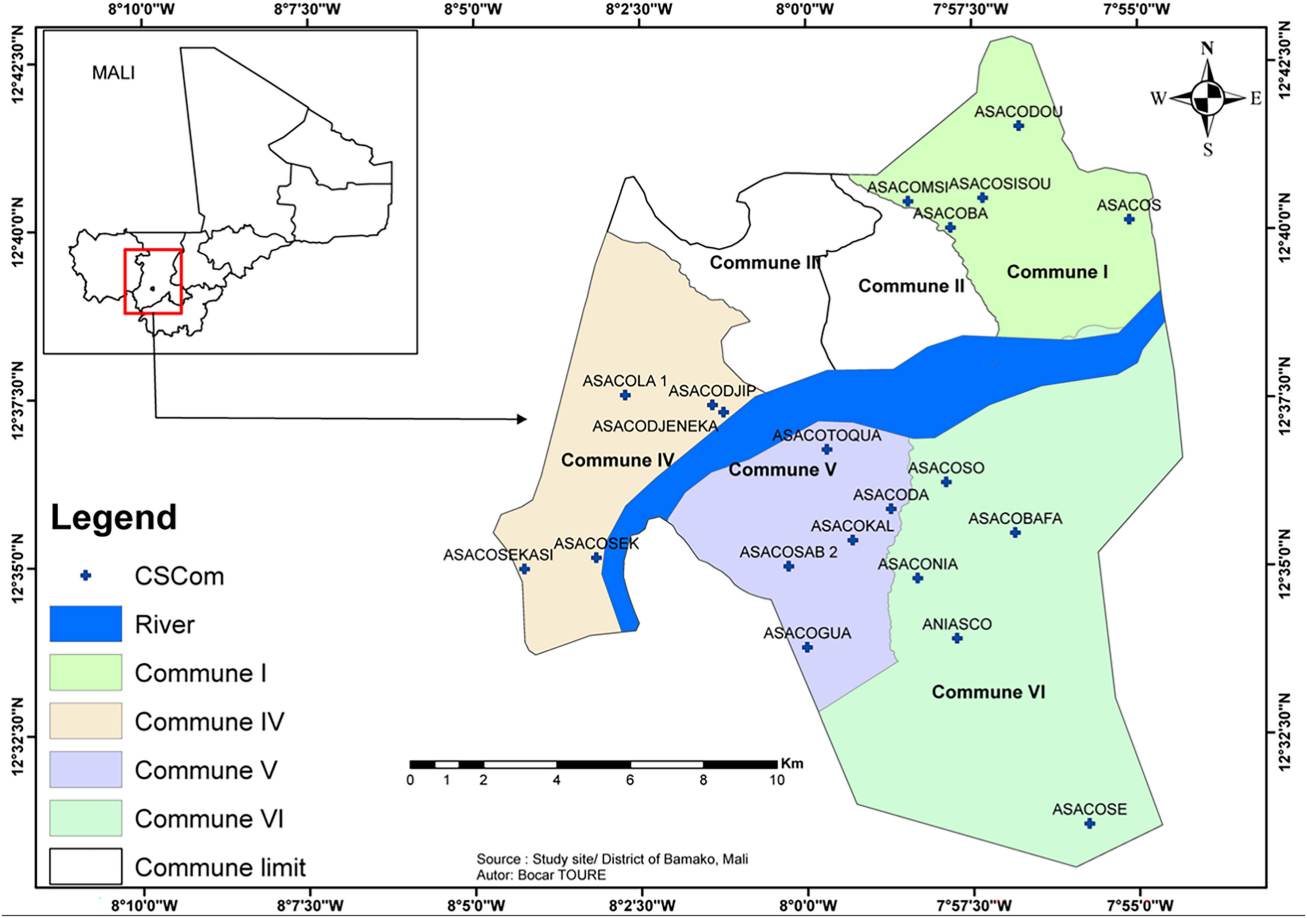
A map of Bamako city with different sites. The map shows the different community health centers (CSComs (+)) in four of the six communes (I, IV, V, and VI) in Bamako. The Niger River intersecting Bamako is shown in blue.

In 2022, the population of Bamako was estimated to be 3 million. Bamako hosts a diverse population composed of different ethnic groups from Mali and neighboring countries. Similar to many African countries, the population of Bamako is predominantly young. This study was conducted in the District of Bamako, the capital of Mali and the epicenter of the COVID‐19 pandemic in the country. The District of Bamako is intersected by the Niger River and is divided into six communes (I–VI) (Figure 1), along with six health districts (HDs). Each HD is divided into community health centers (CSComs). For logistical reasons, we considered two communes from each side of the Niger River of Bamako (i.e., two communes from the right and two communes from the left sides). The CSComs were suggested (by each HD) for their capacity to ensure sample and data collection, as well as for their location allowing easy access to study volunteers. Twenty CSComs (five per communes) were thus selected. Community members were invited to join their CSComs for the enrollment. The selected CSComs included ASACOSISOU, ASACOMSI, ASACOS, ASACOBA, ASACODOU, ASACOSEK, ASACOSEKASI, ASACODJIP, ASACOLA 1, and ASACODJENEKA on the west side of the river and ASACOSAB 2, ASACOKAL, ASACOGUA, ASACODA, ASACOTOQUA, ASACOBAFA, ASACOSE, ANIASCO, ASACONIA, and ASACOSO on the east side (Figure 1).

### 2.3 Sampling and Recruitment

An estimate of the general population size of each commune was derived from the last available census of each HD in Bamako (Table 1). The sample size was calculated based on the general population and national prevalence of COVID‐19 for each age group [19], with a 95% confidence interval, 80% statistical power, and a 10% nonresponse rate. Therefore, the minimum sample size for this study was 3601 participants, accounting for population size and age group (Table 1). Blood samples were collected from all participants. For the recruitment of the participants, together with the community health workers (CHWs, which are responsible for awareness, information, education, and communication activities and most often involved in research or health activities initiated at a community level), the study team invited population to visit the CSComs (Figure 1) to be enrolled. Together with the sociologist, the CHWs passed information regarding the study and the recruitment date in the communities and invited them to attend the CSComs on the appointed date. Indeed, each volunteer was given clear information on the objectives, the ins and outs, scientific benefits for the general community of such a study and gave his/her consent and/or assent. Participants were then recruited exhaustively on a first‐come, first‐served basis. A fixed number of participants were thus drawn from each age group to ensure the age‐stratified information of 1–9, 10–19, 20–29, 30–39, 40–49, 50–59, and ≥ 60 years.

**TABLE 1:** Determining the sample size.

	Commune I	Commune IV	Commune V	Commune VI	Total
	***N*** = 423,637	***N*** = 379,024	***N*** = 523,748	***N*** = 593,976	***N*** = 1,920,385
**Age group (year‐old)**	***n***	***n***	***n***	***n***	***n***
1–9	45	50	28	36	159
10–19	117	140	107	115	479
20–29	207	239	268	236	950
30–39	227	235	271	231	964
40–49	136	117	115	132	500
50–59	85	80	61	78	304
≥ 60	84	43	56	62	245
Total	901	904	906	890	3601

*Note:* The number of participants in each age group was determined for each Commune of Bamako (four communes in total).

Abbreviations: *N*, estimated total population for each commune; *n*, number of enrolled participants for each age group.

### 2.4 Inclusion Criteria

The eligibility criteria were as follows: Participants must be a resident of the selected locality, regardless of age.

### 2.5 Noninclusion Criteria

Children under 1 year of age, persons temporarily living in Bamako, and those suffering from diseases (such as mental and hemorrhagic) were not included.

### 2.6 Data and Sample Collection Procedure

After obtaining the consent of each participant, a questionnaire was administered to collect data about their sociodemographic and clinical backgrounds, whether they had recent exposure to patients with COVID‐19, and their COVID‐19 vaccination status. Information was collected using the Research Electronic Data Capture (REDCap) system (collection, transfer, and systematic review) and R‐Studio version 2023.03.0 for statistical analysis. A trained biologist obtained venous blood samples (4 mL) from each participant using BD Vacutainer venous blood collection tubes and SST Serum Separation Tubes (Becton Dickinson, Franklin Lakes, NJ, USA). Blood samples were transported to the University Clinical Research Center (UCRC) laboratory, where the serum was extracted, aliquoted, and stored in a −80°C freezer until their use in our study. All laboratory‐confirmed COVID‐19 cases were managed systematically according to the national protocol.

### 2.7 SARS‐CoV‐2 Antibody Determination

Antibodies against the trimeric SARS‐CoV‐2 S protein and its RBD were tested at the Malaria Research and Training Center/immunogenetic unit using a reference ELISA, as described previously [20]. Samples were tested in duplicate and standardized by incorporating positive and negative controls, as well as a blank on each plate. The ELISA experiments were optimized using negative Malian control samples. These were used to define baseline antigen recognition [21]. Seropositive responses were defined as samples with OD values above baseline.

### 2.8 Statistical Analysis

Demographic and clinical data were collected using the REDCap system and imported into Microsoft Excel for coding: The R‐Studio version 2023.03.0 was used for the statistical analysis. Participants were categorized by age group. Descriptive analyses were performed to determine the sociodemographic characteristics, vaccine status, exposure factors, and SARS‐CoV‐2 antibody seroprevalence. The chi‐squared (χ^2^) test was used to compare these proportions across different groups with the *p* values calculated using rigorous statistical methods and interpreted at a conventional alpha threshold of 5% to determine statistical significance.

To elucidate factors associated with antibody responses, multivariable logistic regression models (unadjusted and adjusted) were performed, to precisely differentiate anti‐S and anti‐RBD antibody responses as dependent variables. Sex, age group, exposure to COVID‐19 patients, and vaccine status were selected as independent variables, based on their potential influence on COVID‐19. Odds ratios (ORs) were used to quantify the association between antibody positivity and independent variables with a 95% confidence interval. Additionally, a thorough evaluation of model fitness was conducted. This included checks for multicollinearity, assessment of residuals, and verification of model assumptions to ensure robustness and reliability of the findings. Indeed, we employed variance inflation factors (VIFs) to assess multicollinearity among the predictors in the models. The VIF results for both anti‐S and anti‐RBD models showed that all predictors had VIF values significantly less than the commonly used threshold of 5, with the highest VIF recorded at 1.185 for age groups in the anti‐RBD model. These results indicate no concerning multicollinearity, thus affirming the independence of our predictors. Specifically, the VIF for age group, sex, COVID‐19 patient contact, and vaccination status ranged from 1.001 to 1.185, demonstrating that the predictors do not inflate the variance of the regression coefficients due to multicollinearity. For the residual analysis, we meticulously examined the residuals of the models to validate the assumptions required for logistic regression. The residual plots for the anti‐S and anti‐RBD model showed a random dispersion of residuals around the zero line, suggesting that the model assumptions of linearity, independence, and homoscedasticity are adequately met. Altogether, the absence of multicollinearity ensures that our predictors are independent of each other, while a proper residual analysis confirms that the model assumptions are met, indicating reliable and accurate results.

### 2.9 Management of Missing Data

The data for this study was carefully collected as part of a clinical research investigation, and rigorous data collection protocols established by our data management team were followed to ensure that the data was high quality. A continuous validation process for data completeness was implemented by the data management team on the server, allowing us to maintain the proportion of missing clinical data below 5%. These missing data, primarily resulting from unintentional omissions during data entry, can be categorically defined as missing completely at random (MCAR). Again, these occurrences are typical MCAR data. Given the nature (MCAR) and the extremely low proportion of these missing data, there was no need for imputation.

### 2.10 Ethical Considerations

The study protocol was approved by the ethics committee of the University of Sciences, Techniques, and Technologies of Bamako (USTTB) under number 2021/263/USTTB and was authorized by the National Scientific Committee and the Minister of Health. The protocol was presented to local teams at each of the different study sites before beginning the study. All the participants signed an informed consent form. A unique ID was assigned to each participant during enrollment to ensure that their anonymity was preserved during the study.

## 3 Results

### 3.1 Summary of the Sociodemographic and Clinical Characteristics of Study Participants

**FIGURE 2:**
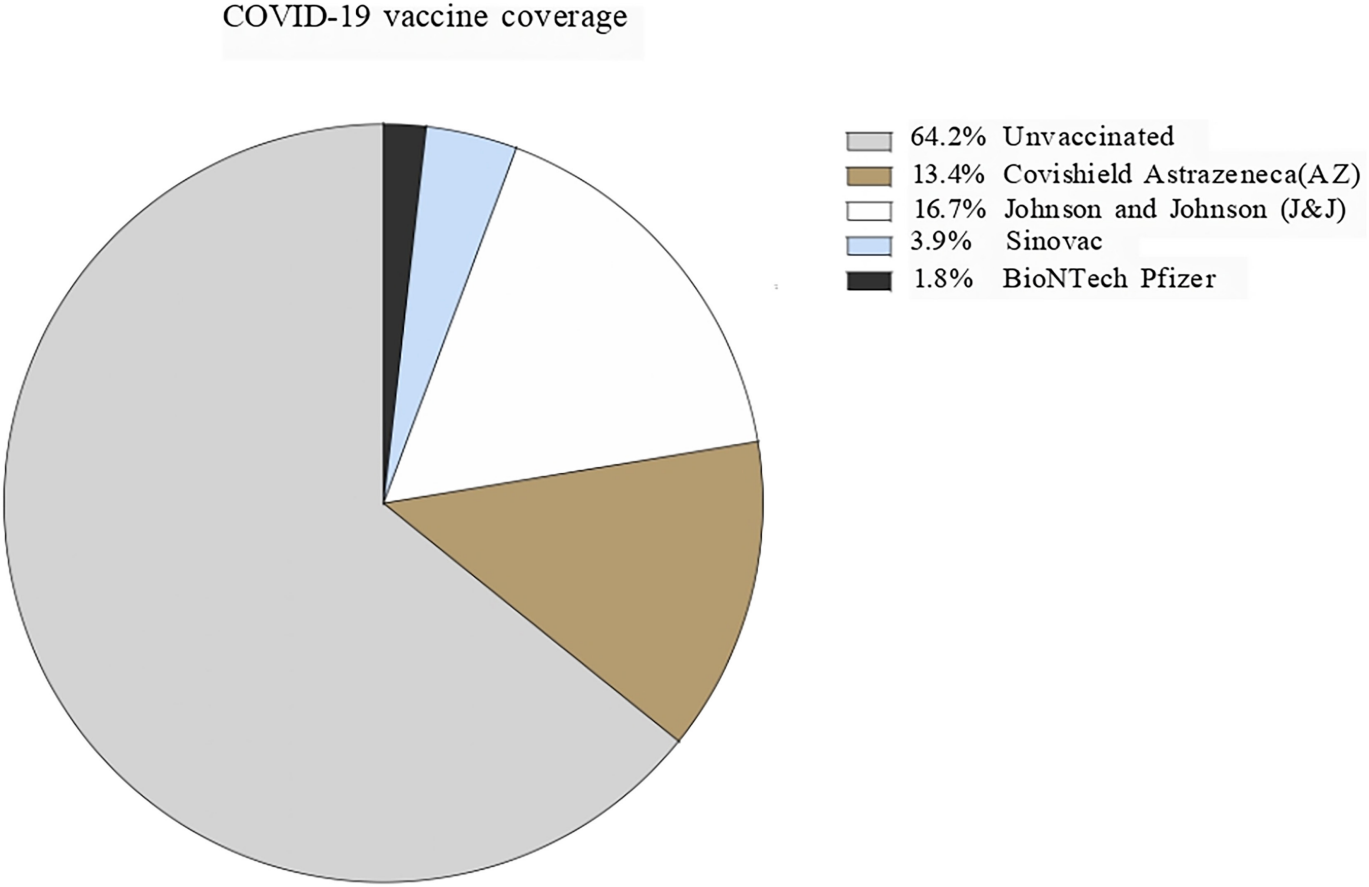
Distribution of participants by types of vaccines. Among the vaccinated group (35.8% of all participants), the coverage rate (%) for each COVID‐19 vaccine was estimated.

A total of 3601 participants were included in the study, with a female‐to‐male ratio of 3.6:1 (Table 2). The ages ranged from 1 to ≥ 60‐year‐old. Furthermore, 20–29 years old (28.9%, *n* = 1043) and 30–39 years old (26.9%, *n* = 967) were the most representative age groups of the study. In terms of occupation, students were the most representative, accounting for 30.0% (*n* = 1065) of the total study group. The second largest group was housewives (25.0%, *n* = 908). The percentage of participants who reported exposure to patients with COVID‐19 was 2.2% (*n* = 80) (Table 2). Among participants, 35.8% (*n* = 1296; Table 2) had received at least one dose of COVID‐19 vaccines, which consisted of the Covishield AstraZeneca (AZ, 13.4%), Johnson & Johnson (J&J, 16.7%), Sinovac (3.9%), and BioNTech Pfizer (1.8%) vaccines (Figure 2). Overall, 98.0% of the collected samples were positive for the S protein and 97.0% for the RBD of SARS‐CoV‐2 (Table 2).

**TABLE 2:** Basic characteristics of the study participants.

Characteristic	Participants (***N*** = 3601) (***n***)	Frequency (%)
**Sex**		
Female	2816	78.0
Male	785	22.0
**Age group (year‐old)**		
01–09	159	4.4
10–19	479	13.0
20–29	950	26.0
30–39	964	27.0
40–49	500	14.0
50–59	304	8.4
≥ 60	245	6.8
**Professions**		
Health worker	369	10.0
Administrator	206	5.7
Commercial agent	528	15.0
Farmer	29	0.8
Housewives	908	25.0
Independent	244	6.8
Student	1065	30.0
Retired	230	6.4
Other	22	0.6
**Exposure to patient with COVID‐19**		
No	3274	90.9
Yes	80	2.2
Unknown	241	6.7
Missing	6	0.2
**Vaccine status**		
Unvaccinated	2296	63.8
Vaccinated	1296	36.0
Unknown	6	0.2
Missing	3	0.1
**Anti‐Spike responder**		
Negative	88	2.4
Positive	3509	97.4
Missing	4	0.1
**Anti‐RBD responder**		
Negative	91	2.5
Positive	3506	97.4
Missing	4	0.1

*Note*: *n* and % represent the number and frequency (percentage) of participants per characteristic, respectively.

Abbreviation: *N*, total number of participants.

### 3.2 Seroprevalence of SARS‐CoV‐2 Antibodies and Factors Impacting Antibody Responses Among the General Population of Bamako

Given that most participants responded to S protein and RBD (98.0% and 97.0%, respectively), we assessed the impact of sociodemographic, clinical, and immunological characteristics on antibody responses. We first studied factors such as sex, age, profession, reported exposure to patients with COVID‐19, and COVID‐19 vaccination status using bivariate analysis (Table 3). Responses to S protein and RBD were significantly higher in female participants (98%) than in male participants (96.0%) (*p* < 0.001) (Table 3). Younger participants (1–9 years old) demonstrated significantly lower antibody responses to S protein and RBD (*p* < 0.0001) than older participants (> 10 years old) (Table 3). Regarding the profession of participants, significant variation in antibody response was observed for RBD (*p* < 0.0001) whereas S showed a slight difference (*p* = 0.0508) (Table 3). Participants with previous exposure to patients with COVID‐19 did not show significantly higher antibody responses against S protein (99.0% vs. 97.0%, *p* = 0.5) or RBD (99.0% vs. 97.0%, *p* = 0.7) than those who had no contact with patients with COVID‐19 (Table 3). Furthermore, participants with one or more COVID‐19 vaccinations demonstrated significantly higher antibody responses against the S protein (98.0% vs. 97.0%, *p* = 0.042) and RBD (99.0% vs. 97.0%, *p* < 0.001) than the unvaccinated participants (Table 3).

**TABLE 3:** Seroprevalence of anti‐S and anti‐RBD antibodies according sociodemographic and clinical characteristics.

Characteristic	Anti‐S responders (***N*** = 3509)	***p*** value	Anti‐RBD responders (***N*** = 3506)	***p*** value
	***n*** (%)		***n*** (%)	
**Sex**		< 0.001		< 0.001
Female	2758 (98)		2756 (98)	
Male	751 (96)		750 (96)	
**Age group (year‐old)**				
01–09	140 (88)	< 0.0001	121 (76)	< 0.0001
10–19	461 (96)		469 (98)	
20–29	928 (98)		936 (99)	
30–39	953 (99)		950 (99)	
40–49	489 (98)		490 (98)	
50–59	299 (99)		300 (99)	
≥ 60	239 (98)		240 (98)	
**Professions**				
Health worker	363 (99)	0.0508	364 (99)	< 0.0001
Administrator	204 (99)		203 (99)	
Commercial agent	521 (99)		523 (99)	
Farmer	29 (100)		29 (100)	
Housewives	885 (98)		890 (98)	
Independent	238 (98)		238 (98)	
Student	1024 (96)		1.012 (95)	
Retired	224 (97)		225 (98)	
Other	21 (95)		22 (100)	
**Exposure to patient with COVID‐19**		0.5		0.7
No	238 (99)		237 (98)	
Yes	3186 (97)		3.184 (97)	
Unknown	79 (99)		79 (99)	
Missing	6		6	
**Vaccine status**		0.042		< 0.001
Unvaccinated	2227 (97)		2217 (97)	
Vaccinated	1273 (98)		1280 (99)	
Unknown	6 (100)		6 (100)	
Missing	3		3	

*Note: n* (%) and number (percentage) of responder proportions for S (Spike) or RBD. Chi‐squared (*χ* ^2^) test was used to compare these proportions across different groups.

Abbreviation: *N*, total number of samples tested in ELISA.

A multivariable logistic regression analysis (adjusted) showed that for both S and RBD (Table 4), the odds of antibody responses against S protein (*p* = 0.024) and RBD (*p* = 0.040) were significantly lower in males than in females (Table 4), confirming the trend observed in bivariate analysis in Table 3. The odds of antibody elucidation were significantly lower at an early age (1–9 years) (*p* < 0.001), increased progressively up to 30–39 years old, declined for the 40–49 years old age group, and increased again with age thereafter (Table 4). Individuals who reported exposure to COVID‐19 did not show significant higher antibody levels against the S protein and RBD. Overall, the trend in S and RDB antibodies in the adjusted analysis remained consistent in the unadjusted model. With regard to vaccination status, COVID‐19 vaccination was associated with a significant increase in antibody response against S protein (*p* = 0.009) in the adjusted model, unlike the unadjusted model (*p* = 0.26). However, for RDB, antibody responses increased significantly in both the adjusted (*p* = 0.042) and unadjusted (*p* < 0.001) models (Table 4).

**TABLE 4:** Multivariable logistic regression analysis of the effects of demographic and clinical characteristics on antibody responses against SARS‐CoV‐2.

Characteristic	Anti‐RDB						Anti‐S					
	Unadjusted			Adjusted			Unadjusted			Adjusted		
	OR	95% CI	***p*** value	OR	95% CI	***p*** value	OR	95% CI	***p*** value	OR	95% CI	***p*** value
**Sex**												
Female	—	—		—	—		—	—		—	—	
Male	0.45	0.29–0.71	< 0.001	0.6	0.37–0.99	0.04	0.46	0.29–0.72	< 0.001	0.58	0.37–0.94	0.024
**Age group (year‐old)**												
01–09	—	—		—	—		—	—		—	—	
10–19	15.7	7.63–35.7	< 0.001	14.7	7.12–33.5	< 0.001	3.62	1.80–7.28	< 0.001	3.42	1.70–6.91	< 0.001
20–29	20.7	11.0–41.0	< 0.001	16.2	8.46–32.5	< 0.001	5.79	2.99–11.1	< 0.001	4.8	2.43–9.41	< 0.001
30–39	22.7	11.9–45.7	< 0.001	16	8.12–33.3	< 0.001	14.6	6.56–34.7	< 0.001	11.1	4.85–27.3	< 0.001
40–49	15	7.44–32.8	< 0.001	10.4	4.97–23.4	< 0.001	6.01	2.80–13.5	< 0.001	4.55	2.04–10.6	< 0.001
50–59	29.7	10.4–125	< 0.001	21.2	7.27–90.7	< 0.001	9.84	3.58–34.6	< 0.001	7.71	2.73–27.6	< 0.001
≥ 60	24.1	8.44–102	< 0.001	18.2	6.24–78.0	< 0.001	7.98	2.90–28.1	< 0.001	6.72	2.38–24.1	< 0.001
**Exposure to patient with COVID‐19**												
No	—	—		—	—		—	—		—	—	
Yes	2.14	0.47–38.0	0.45	1.48	0.31–26.7	0.7	2.09	0.46–37.1	0.47	1.73	0.37–30.8	0.59
**Vaccine status**												
Unvaccinated	—	—		—	—		—	—		—	—	
Vaccinated	3.18	1.85–5.90	< 0.001	1.9	1.05–3.65	0.042	1.97	1.21–3.34	0.009	1.37	0.81–2.39	0.26

*Note:* The association of different factors (age, exposure to patients with COVID‐19, sex, and vaccine status) and the odds ratio (OR) to anti‐S (Spike) and anti‐RBD antibody responses was determined in a multivariable logistic regression analysis (unadjusted and adjusted models).

Abbreviations: OR = odds ratio, CI = confidence interval.

## 4 Discussion

To the best of our knowledge, this is the first study since the rollout of COVID‐19 vaccines to assess the prevalence of antibodies against SARS‐CoV‐2 and analyze the associated factors that may impact antibody responses in the general population of Bamako, the epicenter of the pandemic in Mali. This cross‐sectional study involved 3601 participants from four of the six administrative communes in Bamako, representative of the general population. Our selection incorporated communes on both sides of the Niger River and considered the daily output of the national health surveillance infrastructure, which reported a high prevalence of SARS‐CoV‐2 infection [19]. Participant recruitment was based on the age and population size of each commune to ensure that the age‐specific prevalence of SAR‐CoV‐2 infection was reflected in the study.

Overall, the antibody response prevalence against S protein and RBD was remarkably high in the general population (98.0% and 97.0%, respectively), in contrast to case surveillance (RT‐PCR confirmation) at the national level, which reported approximately 0.0% SARS‐CoV‐2 infection at the time of this study (September 2022) [14]. Overall, our findings are consistent with previous studies conducted in the community (through the healthcare infrastructure, or the blood‐donor center in Mali), all of which showed a higher seroprevalence of SARS‐CoV‐2 antibodies than would be expected from case‐based surveillance [15, 16, 21]. Indeed, antibody seroprevalence increased dramatically from 10.9% to 54.7% in a rural community surveyed in 2021 over an interval of 6 months [21]. Additionally, high antibody levels against COVID‐19 (~70%) were observed in the Koutiala district (southern Mali) in samples collected between June 2020 and January 2021 [16]. In HCW communities, the prevalence of SARS‐CoV‐2 antibodies was high, ranging from 50% to 70% between June 2020 and January 2021 [15]. These findings, in conjunction with our current findings, clearly illustrate a discrepancy between the SARS‐CoV‐2 antibody–based assessment and case‐based surveillance, leading to the conclusion that the magnitude of the pandemic in Mali may be underestimated. Unlike previous studies, this study demonstrated much higher antibody levels against COVID‐19 antigens. This may be due to the cumulative immunity in the population resulting from natural infection but also from COVID‐19 vaccination coverage, which was approximately 36% in this study participants. Furthermore, various studies carried out in different regions or countries of Africa have also confirmed a high seroprevalence of antibodies against SARS‐CoV‐2 in the African populations or cohorts studied [22, 23, 24, 25]. Altogether, these high prevalences of SARS‐CoV‐2 antibodies in Africa should be interpreted with caution due to the cross‐immunities to other vaccinations, tropical diseases (such as malaria), or other SARS infections, all of which may impact on antibody responses against SARS‐CoV‐2 [17, 26, 27, 28]. Further studies are necessary to determine the dynamics of SARS‐CoV‐2 antibodies, the onset of cumulative immunity, and the potentially critical role of cross‐reactive immunity in these communities.

The antibody responses to SARS‐CoV‐2 in this study varied with age and sex. Antibodies against both S protein and RBD were significantly lower in the younger age group of children (1–9 years old) and overall increased with age, despite a sudden drop in antibody levels in the 40–49 years old age group. It has been suggested that children show diminished functional antibody responses in comparison with adults in the COVID‐19 clinical spectrum [29]. It is indeed postulated that children may elicit more efficient immune‐mediated viral clearance (more robust innate immune responses) [30, 31] and demonstrate lower viral receptor (angiotensin‐converting enzyme 2 [ACE2]) expression in airway epithelial cells [32]. Low expression of ACE2 is consistent with lower antibody levels that are correlated with antigen release by the lysis of virus‐infected cells [29, 31]. In addition, the lower antibody levels in young children may be explained by the fact that they are less mobile, and generally unvaccinated in Mali, and therefore unlikely to develop antibodies. We also found that male participants demonstrated less antibody responses to both the S protein and RBD antigens than female participants. Indeed, some studied postulated immune responses against SARS‐CoV‐2 may differ between males and females, thus influencing their ability to recover from severe infection [33, 34, 35, 36, 37]. For example, it has been shown that higher IgG levels in the early phase of COVID‐19 play a critical role in reducing disease severity and mortality in female group [38].

Participants who reported exposure to patients with COVID‐19 did not show significant increase in antibody levels, warranting further studies. As expected, vaccinations against COVID‐19, which coverage was 35.8% in the study participants, were associated with increased antibody responses against RBD, but not against the S protein. The higher levels of antibodies against RBD in the vaccinated group were consistent with the types of vaccines administered in Mali, in which the RBD fragment was the central target epitope. However, SARS‐CoV‐2 antibody prevalence remained also high in the unvaccinated group, implying a significant contribution from naturally acquired antibodies. These data further strengthen previous findings on the high prevalence of SARS‐CoV‐2 antibodies in the Malian population before COVID‐19 vaccines became available [15, 16, 21]. Likewise, our ongoing study has also shown a similarity between naturally and vaccination‐acquired antibody levels over time in the HCW community, although vaccination coverage among HCWs is higher than in the general population (data pending submission), suggesting crucial contribution of naturally (infection) acquired antibodies.

In view of these results, we postulate that further studies of the impact of routine vaccination on the clinical spectrum of COVID‐19, analysis of the seroprevalence of SARS‐CoV‐2 infection among rural populations, and genomics studies of the different variants would contribute to better apprehend the extent and dynamics of SARS‐CoV‐2 infection in Mali.

In conclusion, this study showed a wide variation in antibody levels against SARS‐CoV‐2 as a function of factors such as age, sex, and COVID‐19 vaccination status in the general population of Bamako. The high antibody prevalence, unlike that observed in case‐based survey, indicates that the extent of the pandemic is underestimated in Mali.

## Author Contributions

**Bourama Traoré:** conceptualization, data curation, formal analysis, funding acquisition, investigation, methodology, project administration, supervision, writing–original draft, writing–review and editing. **Merepen A. Guindo:** conceptualization, data curation, formal analysis, funding acquisition, investigation, methodology, supervision, writing–original draft. **Drissa Konaté:** conceptualization, data curation, formal analysis, funding acquisition, investigation, methodology, project administration, supervision, writing–original draft. **Fousseyni Kané:** conceptualization, data curation, formal analysis, funding acquisition, investigation, methodology, software, supervision, writing–original draft. **Nathan C. Incandela:** data curation, formal analysis, validation, writing–review and editing. **Abdouramane Traore:** data curation, formal analysis, investigation. **Salimata Kanté:** formal analysis, investigation. **Mariam Sidibé:** data curation, formal analysis, investigation. **Bourama Keita:** formal analysis, investigation. **Fatoumata Kasse:** data curation, investigation. **Karamoko Tangara:** data curation, investigation. **Dramane Diallo:** investigation, writing–review and editing. **Issoufi Y. Maiga:** data curation, investigation. **Salif Thiam:** data curation, investigation. **Abdourhamane Cisse:** data curation, investigation. **Khatry M. Siby:** conceptualization, data curation, investigation. **Abdoul R. Dicko:** investigation, supervision. **Mariam Goita:** investigation, supervision. **Diakaridia Kone:** investigation, supervision. **Mamadou Diallo:** formal analysis, investigation, supervision. **Modibo Traore:** investigation, supervision. **Yaya I. Coulibaly:** conceptualization, methodology, supervision, writing–review and editing. **Mahamadou Diakité:** conceptualization, investigation, methodology, supervision, writing–review and editing. **Seydou Doumbia:** conceptualization, investigation, methodology, supervision, writing–review and editing. **Housseini Dolo:** conceptualization, data curation, formal analysis, funding acquisition, investigation, methodology, writing–review and editing. **Saidou Balam:** conceptualization, data curation, formal analysis, funding acquisition, investigation, methodology, project administration, resources, software, supervision, validation, visualization, writing–original draft, writing–review and editing.

## Conflicts of Interest

The authors declare no conflicts of interest.

### Peer Review

The peer review history for this article is available at https://www.webofscience.com/api/gateway/wos/peer‐review/10.1111/irv.13343.

## Data Availability

The data that support the findings of this study are available from the corresponding author upon reasonable request.
